# In-Plane Dynamic Crushing Response and Energy Absorption of a Novel Auxetic Honeycomb

**DOI:** 10.3390/ma19040716

**Published:** 2026-02-13

**Authors:** Xin-Liang Li, Bai-Xuan Song, Peng Jia

**Affiliations:** 1 SINOPEC Research Institute of Petroleum Engineering Co., Ltd., Beijing 102206, China; 2 Tianjin Key Laboratory of Modern Engineering Mechanics, School of Mechanical Engineering, Tianjin University, Tianjin 300072, China; cupid@tju.edu.cn; 3 College of Pipeline and Civil Engineering, China University of Petroleum (East China), Qingdao 266580, China; jiapeng2016@upc.edu.cn

**Keywords:** auxetic honeycomb, theoretical mode, finite element method, deformed shape, stress–strain curve, specific energy absorption

## Abstract

A novel auxetic honeycomb (RSSHR) is developed by introducing the arc-shaped structure into the re-entrant star-shaped honeycomb (RSSH). Based on theoretical models and finite element methods, the dynamic crushing responses of RSSH and RSSHR plate (RSSH_P and RSSHR_P) structures are investigated to elucidate the dependence of plateau stress, negative Poisson’s ratio (NPR), deformed shape and specific energy absorption (SEA) on crushing velocity. The stress–strain curves of two types of structures are calculated to analyze configuration–mechanical property relationships. The results exhibit that the plateau stress and SEA of the RSSH_P and RSSHR_P structures increase as the crushing velocity increases. Owing to the stress-mitigating effect of the arc-shaped structure, the RSSHR_P structure exhibits a stronger NPR effect. And the SEA of the RSSHR_P structure is higher than that of the RSSH_P structure. In addition, it is also found that at low crushing velocity, the stress–strain curves of the two structures exhibit three distinct stages: the elastic stage (I), the stress plateau stage (II) and the densification stage (III). During the crushing process, there are three deformed shapes. They are the global deformed shape, the local deformed shape and the layer-by-layer deformed shape.

## 1. Introduction

Engineering materials possessing multiple outstanding physical properties have garnered significant interest from both the scientific and engineering communities [1,2,3]. These outstanding physical properties are intrinsically linked to the microstructure design of engineering materials. Over the past few decades, extensive research has been conducted to elucidate these structure–property relationships [4,5,6,7]. Mechanical metamaterials (MMs) are a class of novel materials featuring exceptional physical properties and designable structures. Through rational microstructure design, MMs can achieve many physical properties which are rarely present in natural substances, such as negative Poisson’s ratio (NPR) [8], negative thermal expansion coefficient [9,10], excellent energy absorption [11,12,13,14,15,16] and so on.

As a type of MM with unique physical properties, NPR material can expand transversely when stretched and contract transversely when compressed [17]. Accordingly, NPR material is also known as auxetic metamaterial (AM) [18,19]. Due to the unique NPR effect and superb energy absorption capacity, AM has been extensively used in the field of biomedicine [20,21], aerospace [22,23], automotive manufacturing [24,25], explosion protection [26], etc. For instance, automotive protective beams fabricated from auxetic metamaterial can effectively mitigate the impact force on vehicles and absorb a large amount of energy, thereby improving vehicle safety [24,25]. Although AM has been deployed in widespread applications, the deformed shapes and energy absorption capacities of AM under crushing loads have not been thoroughly investigated. Therefore, it is imperative to study the deformed shapes, mechanical behaviors and energy absorption mechanisms of AM under crushing loads.

Lots of experimental, theoretical and numerical methods have been employed to study the deformed shapes and mechanical properties of AM under static and impact loading. As early as the 1870s, Voigt [27] first reported findings on NPR materials through the experimental calculation method. He demonstrated that the Poisson’s ratio of pyrite was −1/7. By 1987, Lake et al. [28,29] first designed an artificial NPR structure in foam material. They reported that the Poisson’s ratio of this type of foam material was approximately equal to −0.7. Zhang et al. [30] used experiments, theoretical models and numerical simulations to investigate the effective Poisson’s ratio and the effective Young’s modulus of a novel arc-star-shaped AM. They found that the effective Poisson’s ratio was affected by the geometry coefficients of the AM. Zhang et al. [31] studied the deformed shape and mechanical response of an improved double-arrow AM through experiments, theoretical and numerical methods. They reported that the improved double-arrow AM exhibited buckling deformation and shrinkage deformation during the compression process. Qi et al. [32] adopted theoretical models and numerical simulations to explore the NPR and deformed shapes of a re-entrant circular honeycomb (RCH) which contained double circular arc cell walls. They found that there was an “X” deformed shape in the RCH. Their calculation results demonstrated that the RCH possesses excellent energy absorption capacities. Jiang et al. [33] employed numerical simulations and experiments to design an improved re-entrant honeycomb by combining the conventional re-entrant honeycomb with a tunable stiffness structure. They reported that the specific energy absorption capacity of the improved re-entrant honeycomb was superior to that of the conventional re-entrant honeycomb. Zhang et al. [34] carried out experiments and finite element simulation to study the elasto-plastic deformation behaviors and energy absorption of AMs with an anti-missing-rib lozenge lattice structure. They demonstrated that the anti-missing rib could enhance the stiffness and the superior specific energy absorption capacity of AMs. Song et al. [35] used an experimental method and the finite element method to design a novel auxetic cylindrical metamaterial which consisted of re-entrant star-shaped honeycomb structures. They found that the energy absorption and mechanical resilience of these auxetic cylindrical metamaterials were better than those of traditional AMs which were made up of beam-based lattice structures.

**Figure 1:**
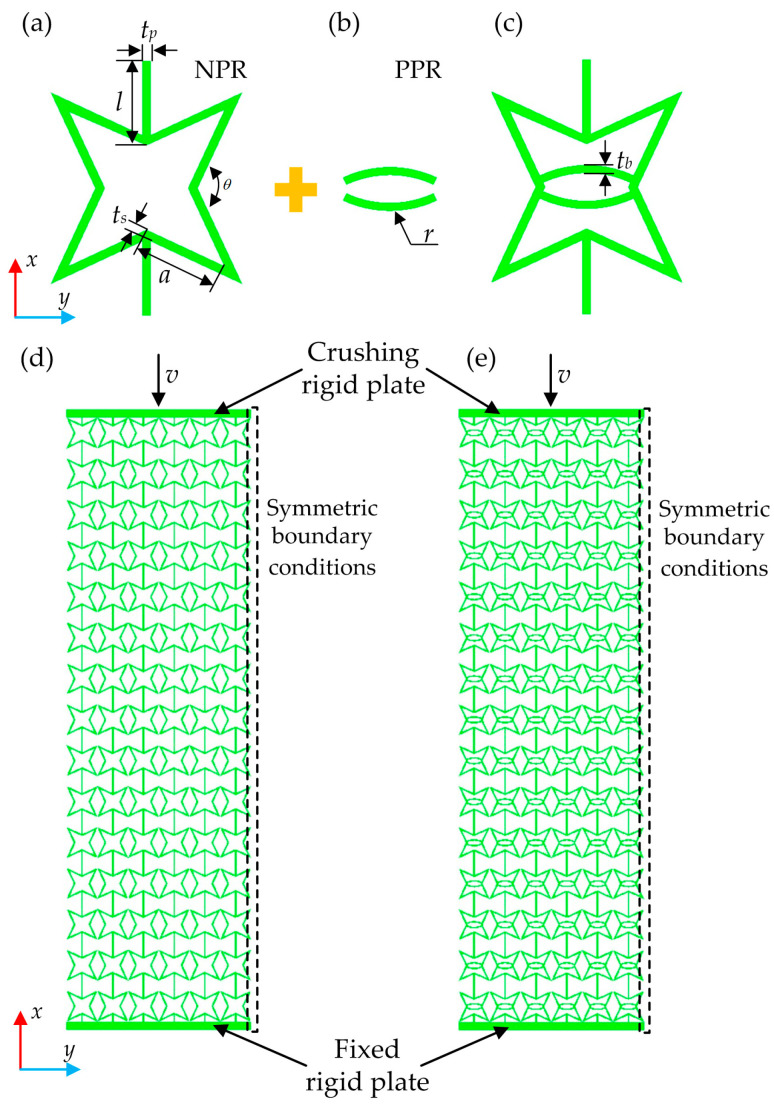
Geometric configurations of auxetic metamaterials. (**a**) RSSH primitive unit cell with NPR, (**b**) arc-shaped structure with PPR, (**c**) RSSHR primitive unit cell, (**d**) RSSH_P structure and (**e**) RSSHR_P structure.

In addition to the mechanical properties under static loading, the deformation shape and energy absorption of AM under crushing loads have also been investigated. Ma et al. [36] adopted theoretical and numerical methods to investigate plateau stress, energy absorption and deformed shape of a hierarchical re-entrant honeycomb AM which consisted of a re-entrant honeycomb structure and a square unit cell. They reported that there was superb specific energy absorption capacity in this type of AM. Zhang et al. [37] used the variable stiffness factor method and the variable energy factor method to explore dynamic mechanical behavior and tunable energy absorption of an AM which consisted of functionally graded auxetic structures. They found that the NPR and energy absorption of the AM could be quantitatively designed by tuning geometrical parameters. The peak force and stiffness ratio of the AM could be reduced by introducing functionally graded auxetic structures. Guo et al. [38] employed finite element simulations to study the deformed shapes and energy absorption of an auxetic cylindrical shell which was made up of a re-entrant auxetic lattice structure. They reported that there was the NPR effect in the auxetic cylindrical shells. Hu et al. [39] used theoretical analysis and numerical simulations to study the NPR effect and dynamic crushing response of AMs which were composed of re-entrant honeycomb structures. They found that the NPR effect of the AMs was significantly influenced by crushing velocity when the compression strain was lower than about 0.2. Qi et al. [40] adopted theoretical models and numerical methods to investigate the deformed shapes and the NPR effect of a tetra-chiral honeycomb AM under in-plane impact loading. They reported that the effective Poisson’s ratio of this type of AM varied with the compression strain. There was a more obvious NPR effect on the AM when the crushing velocity was low. Wang et al. [41] adopted theoretical methods and numerical simulations to study the microstructural deformed shapes and mechanical properties of the re-entrant star-shaped honeycomb (RSSH). They demonstrated that the impact resistance of the RSSH was better than that of the re-entrant honeycomb. In summary, the NPR effect, deformed shape and energy absorption of AMs have been widely investigated. However, the relationships between deformation mechanisms and mechanical properties of auxetic metamaterials—specifically the RSSH and the RSSH with ribs (RSSHR, as shown in Figure 1)—under in-plane crushing remain unclear. Moreover, the influence of crushing velocity on the mechanical response, deformed shape and specific energy absorption has not been thoroughly explored.

In this paper, a novel auxetic metamaterial (the re-entrant star-shaped honeycomb with ribs) is proposed by combining a re-entrant star-shaped honeycomb primitive unit cell and an arc-shaped structure. Based on the theoretical models and finite element methods, the in-plane dynamic crushing behaviors of the RSSH plate structure and the RSSHR plate structure are investigated. The relationships among deformed shapes, mechanical response, specific energy absorption and crushing velocity of the RSSH plate structure and the RSSHR plate structure are elucidated.

## 2. Geometric Configurations and Finite Element Models

### 2.1. Geometric Configurations

The re-entrant star-shaped honeycomb (RSSH) primitive unit cell is a typical auxetic honeycomb primitive unit cell based on the re-entrant mechanism. The RSSH primitive unit cell is made up of square re-entrant corners of thickness *t_s_* and length *a*, joined by straight ribs with length *l* and thickness *t_p_*. *θ* is the angle between the adjacent cell walls, as shown in Figure 1a.

Fu et al. [42] developed a type of MM by combining the re-entrant structure (it possessed NPR) with the rhombus structure (it possessed positive Poisson’s ratio (PPR)). This type of MM exhibited the same mechanical response under tensile loads and compressive loads. Inspired by the above study, this paper combines the RSSH primitive unit cell (it possesses NPR, as shown in Figure 1a) with the arc-shaped structure (it possesses PPR, as shown in Figure 1b) to construct a novel re-entrant star-shaped honeycomb primitive unit cell with rib (RSSHR), as shown in Figure 1c. Under compressive or tensile loading, this novel RSSHR primitive unit cell can maintain deformation capacity. And the arc-shaped structure can prevent further deformation. The RSSHR primitive unit cell not only maintains the NPR effect but also possesses a certain degree of stiffness. Its stiffness is contributed by both the RSSH primitive unit cell and the arc-shaped structure. So, the stiffness of the RSSHR primitive unit cell is improved. Additionally, the NPR of the RSSH primitive unit cell interacts with the PPR of the arc-shaped structure, allowing for the effective regulation of the NPR of the RSSHR primitive unit cell. In the process of deformation, the deformed shape of the RSSHR primitive unit cell is influenced by the arc-shaped structure. The deformation shape can be designed. However, there are some potential problems in the deformation of the RSSHR primitive unit cell. For example, the RSSH primitive unit cell and the arc-shaped structure will be twisted in the out-of-plane direction. Moreover, under complex stress conditions, the RSSH primitive unit cell and arc-shaped structure may not be in contact but rather interlaced. These deformed shapes may result in instability of the RSSHR primitive unit cell. To avoid the above problems, a more complex curve structure can be used instead of the simple arc-shaped structure. The out-of-plane thickness of the RSSHR primitive unit cell can be increased. The constraints can be applied in the out-of-plane direction of the whole primitive unit cell.

The RSSH and RSSHR structures can be obtained by periodically arranging the RSSH and RSSHR primitive unit cells, respectively. The primitive unit cells are arranged 6 times along the *x* direction and 15 times along the *y* direction. When there are certain thicknesses along the *z* direction in the RSSH and RSSHR structures, the RSSH plate (RSSH_P) structure and the RSSHR plate (RSSHR_P) structure can be constructed, as shown in Figure 1d,e, respectively. In this paper, the length of the straight rib wall *l* and the side length of the square re-entrant corner *a* are both 6 mm. The thickness of straight rib *t_p_*, square re-entrant corner *t_s_* and arc-shaped structure *t_b_* is 0.3 mm, and the angle *θ* is 130°. The thickness of the RSSH and RSSHR structures along the *z* direction is set to 5 mm. The radius *r* of arc-shaped structure is set to 2 mm.

In terms of model manufacturing, the geometric complexity of the RSSH_P and RSSHR_P structures, featuring internal re-entrant angles and arc-shaped structures, makes additive manufacturing, particularly laser-based powder bed fusion and selective laser melting, the most direct and feasible fabrication route. These techniques can fabricate such intricate metallic honeycomb structures (especially in metals like aluminum alloys or titanium) layer by layer without assembly.

### 2.2. Finite Element Models

In this study, the COMSOL/explicit commercial software package (version 6.1) is adopted to systematically investigate the dynamic crushing behavior and energy absorption capacity of the RSSH_P and RSSHR_P structures. As shown in Figure 1, the finite element models of RSSH_P and RSSHR_P structures are located between a crushing rigid plate and a fixed rigid plate. The fixed rigid plate is completely fixed and the crushing rigid plate impacts the honeycomb structure along the *x* direction at constant crushing velocity, as shown in Figure 1. To improve computational efficiency, a half-model is employed by exploiting geometric symmetry. Symmetry boundary conditions are applied on the *y*-normal face (the plane at *y* = 0), which ensures that the mechanical response of the half-model replicates that of the whole structure. To comprehensively investigate how different crushing velocities affect the mechanical response and energy absorption mechanisms, the crushing velocities *v* vary from 1 m/s to 150 m/s. During the calculation process, an elastic–perfectly plastic constitutive relation is adopted for the base material. The base material of honeycomb is aluminum alloy AA6060 T4. It is modeled as rate-independent material with a density of 2700 kg/m^3^, a Young’s modulus of 68.2 GPa, a Poisson’s ratio of 0.3 and a yield stress of 80 MPa [43]. In the models, four-node shell elements are employed to mesh the honeycomb structures in order to enhance computational efficiency. A global element size of 1 mm is adopted, yielding approximately 103,364 elements. This mesh density is determined to be sufficient through a mesh convergence study. The contact pairs are defined between the crushing rigid plate and the honeycomb structure, as well as between the fixed rigid plate and the honeycomb structure. To simulate the complex interfacial interactions, self-contact is enabled for the RSSH_P and RSSHR_P structures. The tangential contact behavior is simulated using a penalty formulation, while the normal contact constraint is enforced via an augmented Lagrangian method. The friction coefficient is set to 0.2. To ensure a plane strain condition in each model, the out-of-plane displacement of all nodes is constrained, while the in-plane movements of all nodes are allowed.

## 3. Verification of Crushing Model Validity

According to previous studies [39,40,44,45], the stress–strain curves of honeycomb structures exhibit a plateau region as the compression strain increases. In this plateau region, the stress fluctuates around a near-constant value. The plateau region and the near-constant value are called the stress plateau stage and the plateau stress, respectively. The plateau stress is an important indicator for evaluating the crushing resistance of the structure. And $\sigma_{p}$ can be obtained by Equation (1).

(1) $$\sigma_{p}=\frac{\int_{\varepsilon_{\mathrm{cr}}}^{\varepsilon_{D}}\sigma (\varepsilon )d\varepsilon }{\varepsilon_{D}-\varepsilon_{\mathrm{cr}}}$$

where $\varepsilon_{D}$ represents the densification strain. $\varepsilon_{\mathrm{cr}}$ corresponds to the initial peak stress of the honeycomb structure. And it is also known as the initial strain. σ(ε) and ε denote stress and strain in the stress–strain curves, respectively. The stress σ(ε) can be calculated as the reaction force on the crushing rigid plate divided by the original cross-sectional area of the honeycomb structure. The strain ε can be obtained from the ratio of the displacement increment of the honeycomb structure along the *x* direction to its original width.

To verify the accuracy of the loading configuration and boundary conditions in the simulations, this paper first calculates the plateau stress of the RSSH_P structure (whose geometry is shown in Figure 1d). The calculation results of this paper are then compared with those of a previous study [46]. In order to improve computational efficiency, a symmetric model of the RSSH_P structure is adopted. And the RSSH_P structure is modeled using shell elements. The symmetric boundary conditions (as described in Section 2) are applied. In addition to the above, all other simulation settings remain consistent with the literature [46].

**Figure 2:**
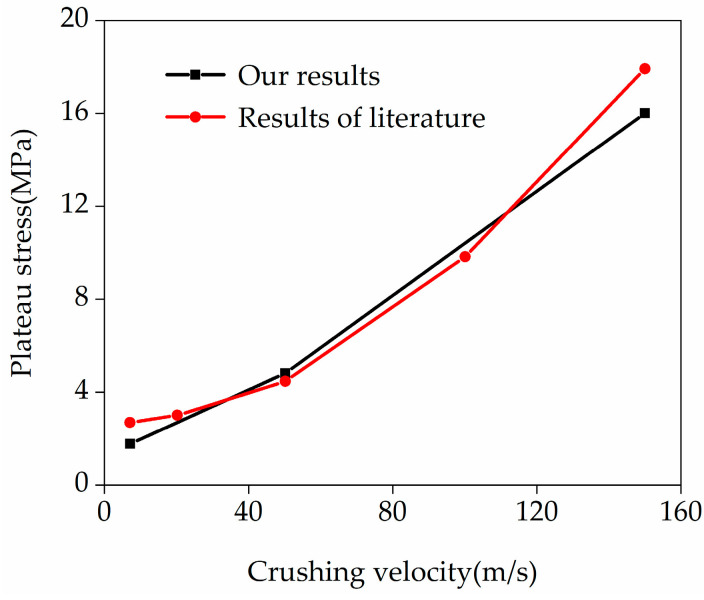
The calculation results in this paper are compared with those from the literature [46].

Figure 2 shows the comparison between the plateau stresses calculated in this paper (different crushing velocities) and those in the literature. The black curve and red curve represent the results from the present finite element simulations and from the literature [46], respectively. It can be seen from the figure that the plateau stress increases progressively with crushing velocity. Moreover, the calculation results of this paper are in excellent agreement with those in the literature, which indicates that the calculation models in this paper are reasonable and reliable.

## 4. The NPR Effects of RSSH_P and RSSHR_P Structures

The NPR effects of RSSH_P and RSSHR_P structures have attracted increasing attention from researchers. In this paper, the NPRs of the RSSH_P and RSSHR_P structures are calculated and compared. In the process of calculation, the ratio of the displacement increment of the RSSH_P structure along the *y* direction to the initial width of the RSSH_P structure is defined as the strain $\varepsilon_{y}$ of the RSSH_P structure along the *y* direction. The ratio of the displacement increment of the crushing rigid plate along the *x* direction to the initial length of the RSSH_P structure is defined as the strain $\varepsilon_{x}$ of the RSSH_P structure along the *x* direction. The Poisson’s ratio of the RSSH_P structure can be obtained through the equation $\upsilon =-(\varepsilon_{y}/\varepsilon_{x})$. Similarly, the Poisson’s ratio of the RSSHR_P structure can be obtained.

**Figure 3:**
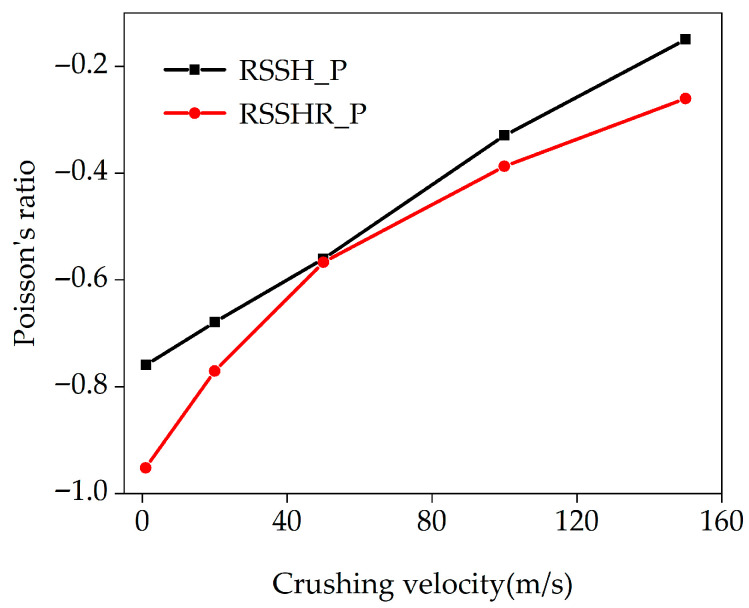
Poisson’s ratio of RSSH_P and RSSHR_P structures under different crushing velocities.

Figure 3 exhibits the relationships between crushing velocities and the Poisson’s ratios of the RSSH_P and RSSHR_P structures. From Figure 3, it can be seen that there is an obvious NPR effect (the Poisson’s ratio is negative) in the two types of structures. When the crushing velocity is low, the two types of structures gradually deform. The RSSH and RSSHR primitive unit cells can fully rotate and deform, leading to significant transverse shrinkage deformations of the RSSH_P and RSSHR_P structures. Therefore, the Poisson’s ratios of two types of structures reach their most negative values (the absolute value of Poisson’s ratio is the largest). The more negative the Poisson’s ratio, the stronger the NPR effect. As the crushing velocity increases, the calculated Poisson’s ratios of the RSSH_P and RSSHR_P structures become less negative. The distances between the Poisson’s ratio curves for the two types of structures first decrease and then increase. When the crushing velocity is high, the inertial effect becomes more conspicuous. The RSSH and RSSHR primitive unit cells are crushed under the circumstance of no complete deformation. The transverse shrinkage deformations of the RSSH_P and RSSHR_P structures are reduced. Therefore, the absolute values of the Poisson’s ratios of the RSSH_P and RSSHR_P structures decrease. The NPR effects are weakened. In addition, we also find that the NPR effect of the RSSHR_P structure is significantly stronger than that of the RSSH_P structure. At crushing velocities of 20 m/s and 100 m/s, the NPRs of the RSSHR_P structure are 13.5% and 17.5% lower than those of the RSSH_P structure, respectively. This enhancement is directly attributed to the unique role of the arc-shaped structure within the RSSHR primitive unit cell. In the process of deformation, the re-entrant region in the RSSHR primitive unit cell gradually engages the arc-shaped structure, and friction arises between them. The arc-shaped structure can effectively alleviate stress concentration in the RSSHR primitive unit cell (detailed analyses can be seen in Section 5.2). Therefore, stable load transfer and reduced stress concentration allow the RSSHR primitive unit cell to undergo more uniform and complete deformations, thereby enhancing the NPR effect of the RSSHR_P structure.

In addition to the relationship between crushing velocity and the Poisson’s ratio, the influence of the radius of the arc-shaped structure on the Poisson’s ratio of the RSSHR_P structure is studied at a crushing velocity of 10 m/s. It is found that as the radius increases, the absolute value of the Poisson’s ratio of the RSSHR_P structure decreases, as shown in Supplementary Figure S1.

## 5. Mechanical Response and Deformed Shapes

According to previous studies [38,39,40], the energy absorption capacity and mechanical response of AMs are strongly dependent on the deformed shapes. Given the diverse deformed shapes of RSSH_P and RSSHR_P structures under crushing loads, it is valuable to explore how these deformed shapes, along with the mechanical response, relate to the crushing velocity for the two types of structures.

### 5.1. Critical Crushing Velocity

According to previous studies [39,40,44], the deformed shapes of honeycomb structures are significantly influenced by the crushing velocity. When the crushing velocity is lower than the first critical velocity *V*_cr1_ (*v* < *V*_cr1_; it is called low-velocity crushing) [47], the honeycomb structure exhibits a global deformed shape. When the crushing velocity is between the first critical velocity *V*_cr1_ and the second critical velocity *V*_cr2_ (*V*_cr1_ < *v* < *V*_cr2_; it is called medium-velocity crushing) [48], the honeycomb structure shows local and global deformed shapes in the first half and the latter half of the crushing process, respectively. When the crushing velocity is greater than the second critical velocity *V*_cr2_ (*v* > *V*_cr2_; it is called high-velocity crushing), the honeycomb structure exhibits a layer-by-layer deformed shape.

The RSSH_P and RSSHR_P structures are honeycomb structures. The first critical velocity *V*_cr1_ can be expressed by Equation (2) [47,49].

(2) $$V_{\mathrm{cr}1}=\int_{0}^{\varepsilon_{\mathrm{cr}}}\sqrt{\frac{\sigma^{\prime }(\varepsilon )}{\rho^{*}}}d\varepsilon$$

where $\varepsilon_{\mathrm{cr}}$ is the initial strain. σ(ε) is the stress. $\sigma^{\prime }(\varepsilon )$ is the first-order derivative of stress. It represents Young’s modulus in the linear stage of the stress–strain curve of the honeycomb structure. $\rho^{*}$ is the density of the honeycomb structure.

When the crushing velocities of the RSSH_P and RSSHR_P structures are greater than the second critical velocity *V*_cr2_, two types of honeycomb structures exhibit a layer-by-layer deformed shape. The second critical velocity *V*_cr2_ can be expressed as [48]

(3) $$V_{\mathrm{cr}2}=\sqrt{\frac{2\sigma_{p}\varepsilon_{D}}{\rho^{*}}}$$

where $\sigma_{p}$ and $\varepsilon_{D}$ represent the plateau stress and the densification strain of the honeycomb structure, respectively.

In this paper, the *V*_cr1_ and *V*_cr2_ of the RSSH_P structure and RSSHR_P structure are calculated by Equations (2) and (3). For the RSSH_P structure, *V*_cr1_ = 38 m/s, and *V*_cr2_ = 110 m/s; for the RSSH_P structure, *V*_cr1_ = 13 m/s, and *V*_cr2_ = 83 m/s. In order to explore the relationships among deformed shapes, mechanical response and the crushing velocity, the crushing velocity of the crushing rigid plate is set to 1 m/s, 20 m/s, 50 m/s, 100 m/s and 150 m/s. Supplementary Figures S2 and S3 present the corresponding stress–strain curves for the RSSH_P and RSSHR_P structures. For simplicity, the deformed shapes of the two types of structures are discussed at representative crushing velocities of 1 m/s, 50 m/s and 150 m/s.

### 5.2. Low-Velocity Crushing

**Figure 4:**
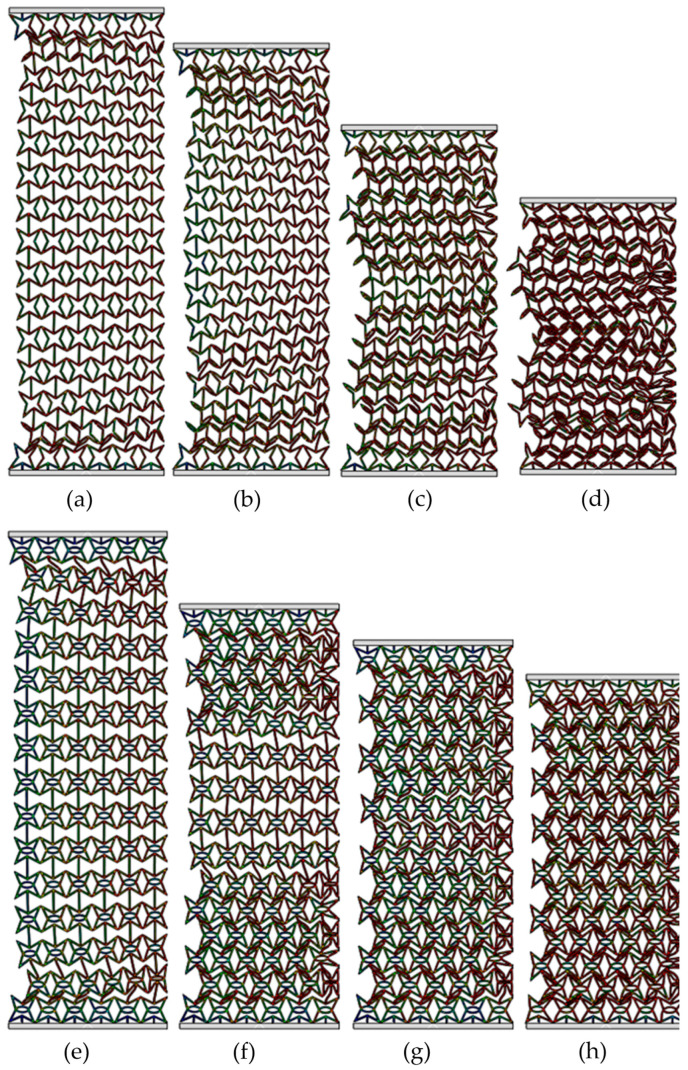
Deformed shapes of RSSH_P and RSSHR_P structures when crushing velocity *v* is equal to 1 m/s. (**a**–**d**) Deformed shapes of RSSH_P structure when compression strains are 0.05, 0.2, 0.4 and 0.6. (**e**–**h**) Deformed shapes of RSSHR_P structure when compression strains are 0.05, 0.2, 0.4 and 0.6.

Figure 4 exhibits the deformed shapes of the RSSH_P and RSSHR_P structures under low-velocity crushing. The crushing velocity *v* is equal to 1 m/s. It can be seen from the figure that when the RSSH_P structure is subjected to crushing load, the deformation bands form simultaneously in the regions adjacent to the crushing end and the fixed end, as shown in Figure 4a. With the increase in compression strain, the primitive unit cells around the deformation bands are gradually crushed. And the crushed primitive unit cells gradually accumulate, as shown in Figure 4b,c. The RSSH_P structure exhibits a global deformed shape. And then as the compression strain further increases, the whole structure gradually densifies, as shown in Figure 4d.

Figure 4e–h show that the deformed shapes of the RSSHR_P structure are almost similar to those of the RSSH_P structure. Because the crushing velocity is low, the inertial effect of the RSSHR_P structure is small. So, there is also a global deformed shape in the RSSHR_P structure. In addition, it is also found that during the deformation process of the RSSHR_P structure, “S”-shaped deformation bands form at the crushing end and the fixed end, as shown in Figure 4f. These deformation bands cause the RSSHR_P structure to contract inward. Thus, the NPR effect of the RSSHR_P structure is enhanced.

**Figure 5:**
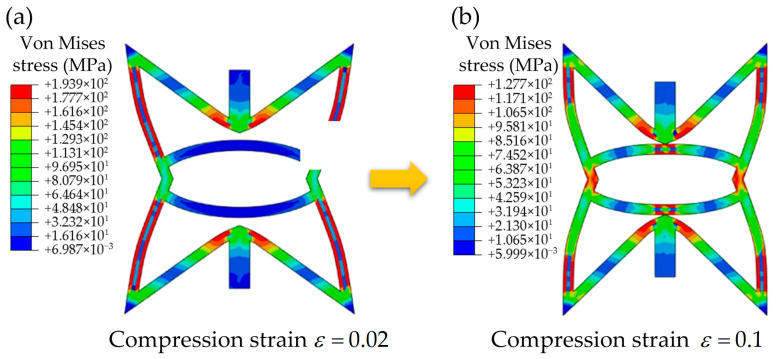
The von Mises stress contour plots of the RSSHR primitive unit cell at (**a**) a compressive strain of ε =0.02 and (**b**) a compressive strain of ε =0.1. The labeled color scale bar (legend) represents the magnitude of the von Mises stress of the RSSHR primitive unit cell.

In addition to the above, the interactions between the RSSH region and the arc-shaped structure within RSSHR primitive unit cell are also explored. Figure 5a,b show the von Mises stress contour plots of the RSSHR primitive unit cell at compressive strains of ε =0.02 and ε = 0.1, respectively. This representative unit cell is selected from the central region of the RSSHR_P structure. From Figure 5a we can see that at a compressive strain of 0.02, the von Mises stress of the RSSH region in the RSSHR primitive unit cell is significantly higher than that of the arc-shaped structure. The stress concentration in the RSSH region reaches 177.7–193.9 MPa (red region), whereas it remains below 32.3 MPa (blue region) in the arc-shaped structure. It can be concluded that the RSSH region in the RSSHR primitive unit cell bears the main loads, while the arc-shaped structure bears almost no load. With further deformation of the RSSHR primitive unit cell, the RSSH region eventually contacts the arc-shaped structure. As a result, the arc-shaped structure begins to participate in load bearing, leading to a more uniform stress distribution. The stress contour plot in Figure 5b demonstrates that at a compression strain of 0.1, the stress concentration of the RSSH region in the RSSHR primitive unit cell decreases to approximately 117.1–127.7 MPa. Accordingly, the incorporation of the arc-shaped structure effectively mitigates stress concentration in the RSSHR primitive unit cell by providing an alternative load path. This optimized stress distribution is a key structural feature that contributes to the improved mechanical performance of the RSSHR_P structure.

**Figure 6:**
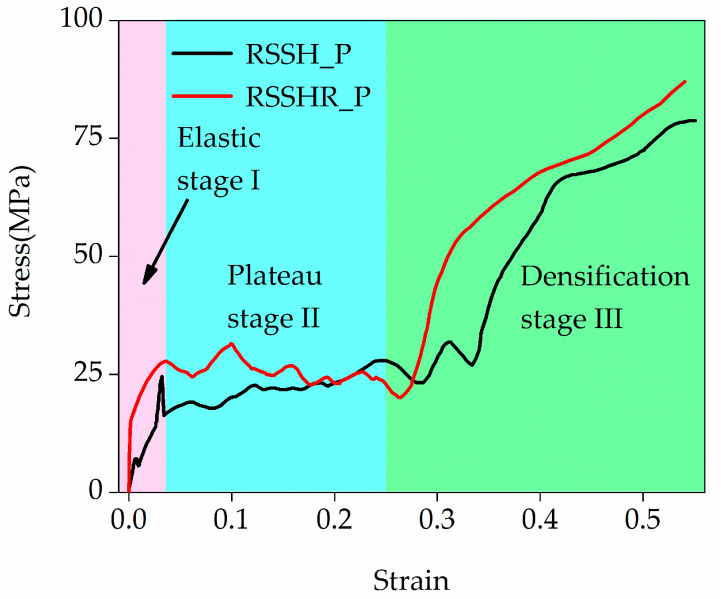
The strain–stress curves of the RSSH_P and RSSHR_P structures when the crushing velocity is equal to 1 m/s.

Figure 6 illustrates the stress–strain curves of the RSSH_P and RSSHR_P structures at a crushing velocity of 1 m/s. During the calculation process, the stress in Figure 6 is obtained by the ratio of the reaction force on the crushing rigid plate to the original cross-sectional area of the honeycomb structure. Crushing is considered complete when all primitive unit cells in the two structures are fully crushed. From Figure 6 we can see that when the crushing velocity is low, the inertial effects of two types of structures are small. And the two types of structures exhibit global deformed shapes. Their stress–strain curves are close to those under static loading. The initial peak stress in the stress–strain curve is not pronounced. Additionally, from Figure 6 we also find that the stress–strain curve of the RSSHR_P structure is higher than that of RSSH_P structure. In this paper, the stress–strain curve of the RSSHR_P structure is taken as an example for investigation. The stress–strain curve of the RSSHR_P structure first undergoes an elastic stage (stage I). In this stage, the stress increases linearly with the increase in the compression strain. When the stress of the RSSHR_P structure reaches the initial peak stress (it is not obvious), the stress begins to decrease. The stress–strain curve enters the stress plateau stage (stage II). In this stage, the primitive unit cells are gradually crushed. The stress fluctuates around a near-constant value (it is plateau stress) with the increase in the compression strain. Then as the compression strain further increases, the stress–strain curve enters the densification stage (stage III). In this stage, the whole structure is gradually densified. The stress increases sharply.

### 5.3. Medium-Velocity Crushing

**Figure 7:**
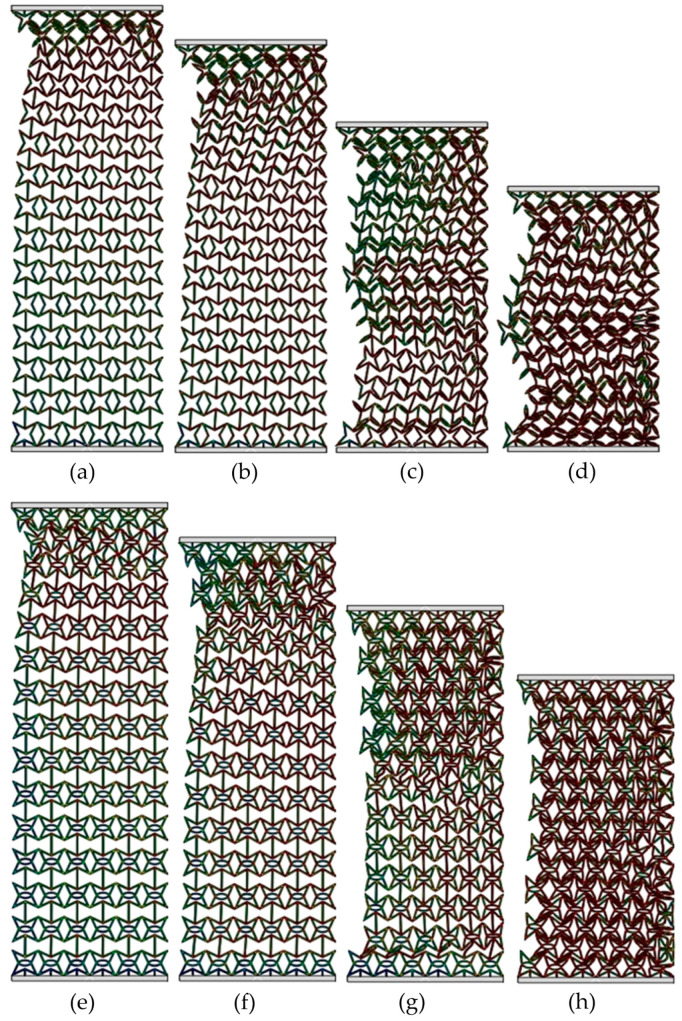
Deformed shapes of RSSH_P and RSSHR_P structures when crushing velocity is equal to 50 m/s. (**a**–**d**) Deformed shapes of RSSH_P structure when compression strains are 0.05, 0.2, 0.4 and 0.6. (**e**–**h**) Deformed shapes of RSSHR_P structure when compression strains are 0.05, 0.2, 0.4 and 0.6.

Figure 7 exhibits the deformed shapes of the RSSH_P and RSSHR_P structures under medium-velocity crushing. The crushing velocity *v* is equal to 50 m/s. Compared with the crushing velocity *v* = 1 m/s, the inertial effects of the two types of structures gradually become apparent due to the increased crushing velocity. It can be seen that the deformation bands will be first formed at the crushing end of the RSSH_P structure, as shown in Figure 7a. Then as the compression strain increases, the thickness of the deformation band gradually increases. And the deformation band gradually spreads from the crushing end to the fixed end. The RSSH_P structure then exhibits a local deformed shape in the regions adjacent to the crushing end, as shown in Figure 7b. Because the crushing velocity is not very high, the inertial effects remain limited, allowing the RSSH_P structure to subsequently exhibit a global deformed shape in the latter half of the crushing process. And then the RSSHR_P structure gradually densifies as the compression strain further increases, as shown in Figure 7c,d. It can be seen from Figure 7e,f that in the first half of crushing process, the deformed shape of the RSSHR_P structure is similar to that of the RSSH_P structure. That is, the deformation band is first formed at the crushing end. Then the thickness of the deformation band gradually increases. And it gradually propagates towards the fixed end. The RSSHR_P structure shows a local deformed shape. The difference is that in the latter half of the crushing process, the deformation band first forms at the fixed end. Then as the compression strain increases, the RSSHR_P structure exhibits a global deformed shape. And then the RSSHR_P structure is completely densified, as shown in Figure 7g,h. This deformed shape enables each layer to deform more thoroughly, thereby allowing the RSSHR_P structure to absorb more energy.

**Figure 8:**
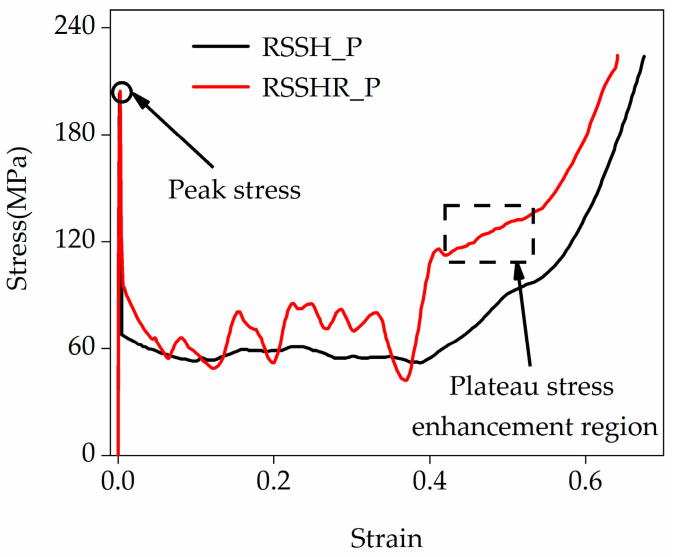
The strain–stress curves of the RSSH_P and RSSHR_P structures when the crushing velocity is equal to 50 m/s.

Figure 8 illustrates the stress–strain curves of the RSSH_P and RSSHR_P structures when the crushing velocity is equal to 50 m/s. It can be seen from Figure 8 that after the onset of crushing, there are the obvious peak stresses in the stress–strain curves of the two types of structures (it is related to local deformation, as shown in Figure 7). Then the stress decreases with the increase in compression strain. The stress–strain curves enter the stress plateau stage. In this stage, the primitive unit cell is gradually crushed. The deformation band gradually spreads from the crushing end to the fixed end. The stress fluctuates around a near-constant value. And then as the compression strain further increases, the stress–strain curves enter the densification stage. In this stage, the whole structure is gradually densified. The stress increases sharply. Interestingly, the more complex deformed shape and initial configuration of the RSSHR_P structure generate greater contact friction during the crushing process. Therefore, the stress–strain curve of the RSSHR_P structure exhibits an additional plateau stress enhancement region (according to the red curve, the strain range is 0.42 to 0.5, and the stress range is 112.5 MPa to 131.3 MPa, as shown in Figure 8) between the plateau stage and the densification stage. In this enhancement region, the stress–strain curve of the RSSHR_P structure encompasses a larger area, enabling it to absorb significantly more energy than the RSSH_P structure. Moreover, the plateau stress remains relatively stable. This stability is crucial when the RSSHR_P structure is used as a protective device, as it ensures smooth deceleration of the protected object. Additionally, it can be seen from the figure that as the crushing velocity increases, the plateau stress of the two types of structures increases. The stress–strain curve of the RSSHR_P structure is higher than that of the RSSH_P structure.

### 5.4. High-Velocity Crushing

**Figure 9:**
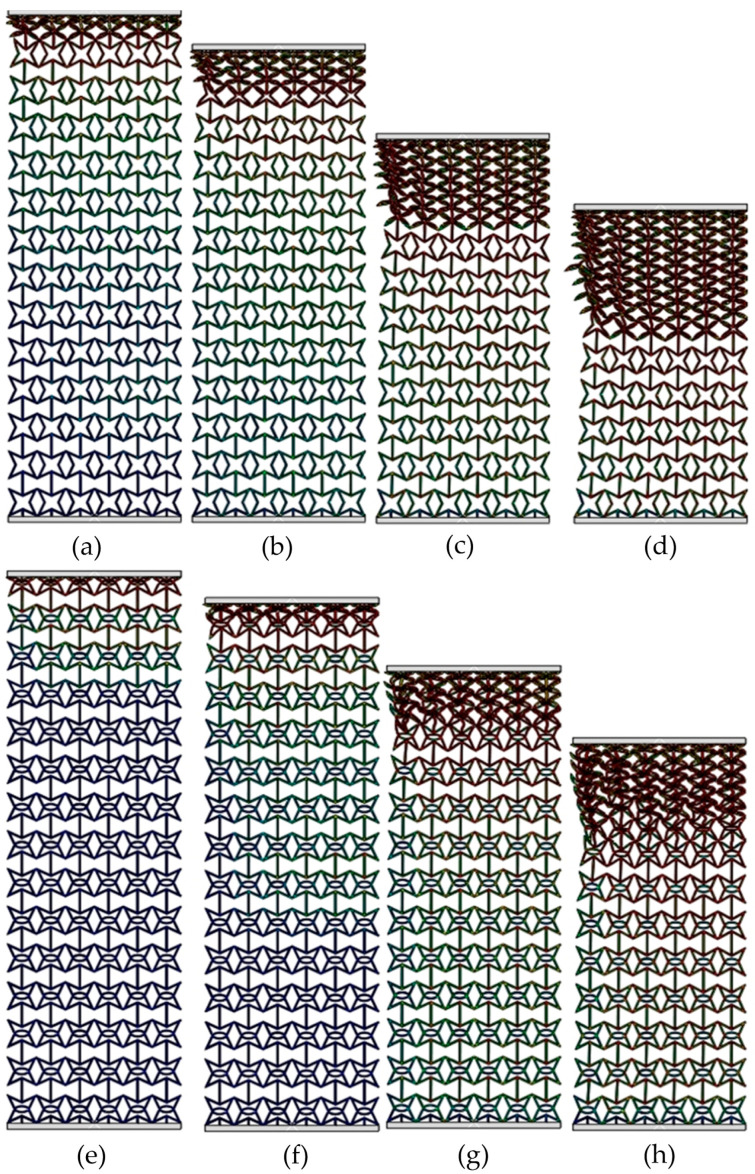
Deformed shapes of RSSH_P and RSSHR_P structures when velocity *v* is equal to 150 m/s. (**a**–**d**) Deformed shapes of RSSH_P structure when compression strains are 0.05, 0.2, 0.4 and 0.6. (**e**–**h**) Deformed shapes of RSSHR_P structure when compression strains are 0.05, 0.2, 0.4 and 0.6.

Figure 9 shows the deformed shapes of the RSSH_P and RSSHR_P structures under high-velocity crushing. It can be seen from Figure 9 that the inertial effects of the RSSH_P structure and RSSHR_P structure become very obvious when the crushing velocity *v* is 150 m/s (it is greater than *V*_cr2_). After the onset of crushing, the primitive unit cells adjacent to the crushing ends of the two types of structures are rapidly crushed due to the high crushing velocity. And an “I”-shaped deformation band is formed, as shown in Figure 9a,e. Then as the compression strain increases, the thickness of the “I”-shaped deformation band gradually increases. The “I”-shaped deformation band spreads layer by layer from the crushing end to the fixed end, as shown in Figure 9b,c,f,g. The two types of structures exhibit layer-by-layer deformed shapes. And then as the compression strain further increases, the RSSH_P and RSSHR_P structures gradually densify, as shown in Figure 9d,h. In addition to the above analyses, it is also found from Figure 9e,h that when the crushing velocity is high, the “S”-shaped deformation band of the RSSHR_P structure is absent. The reason is that the high crushing velocity suppresses the transverse shrinkage of the primitive unit cells at the shock wave front. Consequently, the RSSHR_P structure under high-velocity crushing conditions fails to demonstrate a significant dynamic negative Poisson’s ratio, as shown in Figure 3.

**Figure 10:**
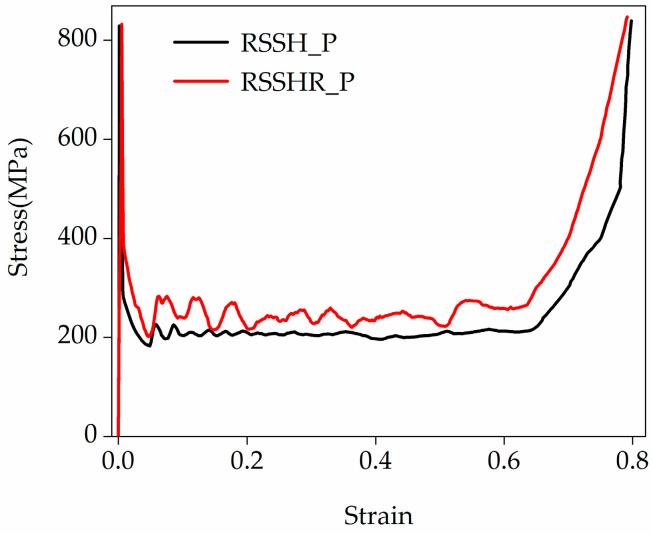
The strain–stress curves of the RSSH_P and RSSHR_P structure when the crushing velocity is equal to 150 m/s.

Figure 10 illustrates the stress–strain curves of the RSSH_P and RSSHR_P structures under high-velocity crushing (the crushing velocity is equal to 150 m/s). From Figure 10 we can see that after the onset of crushing, the stress–strain curves of the two types of structures first exhibit the extremely high peak stresses (it is related to the “I”-shaped deformation band, as shown in Figure 9). Then as the compression strain increases, the stress decreases rapidly. The stress–strain curves enter the stress plateau stage. In this stage, the “I”-shaped deformation band spreads layer by layer from the crushing end to the fixed end. The stress fluctuates around a near-constant value. And then as the compression strain further increases, the stress–strain curves enter the densification stage. In this stage, the whole structure is gradually densified. The stress increases sharply. From Figure 9 and Figure 10 we can see that due to the complex initial configurations and deformed shapes, the stress–strain curve of the RSSHR_P structure is higher than that of the RSSH_P structure. And the RSSHR_P structure can absorb more energy. The high crushing velocity leads to the formation of “I”-shaped deformation bands that propagate layer by layer (as shown in Figure 9), which induces pronounced fluctuations in the plateau stage. This is particularly evident in the more obvious stress fluctuations of the RSSHR_P structure (the red curve shown in Figure 10). In addition, it can be observed that when the crushing velocity is high, the plateau stress enhancement region in the stress–strain curve of the RSSHR_P structure is completely absent.

## 6. Energy Absorption Capacity of the Two Structures

The RSSH_P and RSSHR_P structures can absorb a substantial amount of energy because of the special deformation mechanisms and excellent mechanical properties. In recent years, extensive research has been conducted on the energy absorption capacity of RSSH_P and RSSHR_P structures. Specific energy absorption (SEA) is a key parameter for evaluating the energy absorption capacity. It can be expressed as [36]

(4) $$U_{\mathrm{SEA}}=\frac{U_{\mathrm{EA}}}{m}=\frac{\int_{0}^{\varepsilon_{D}}\sigma (\varepsilon )\;d\varepsilon }{\rho^{*}}$$

where $U_{\mathrm{EA}}$ is the total energy absorption, *m* is the mass of the structures, $\varepsilon_{D}$ represents the densification strain, and $\rho^{*}$ is the density of the honeycomb structure.

**Figure 11:**
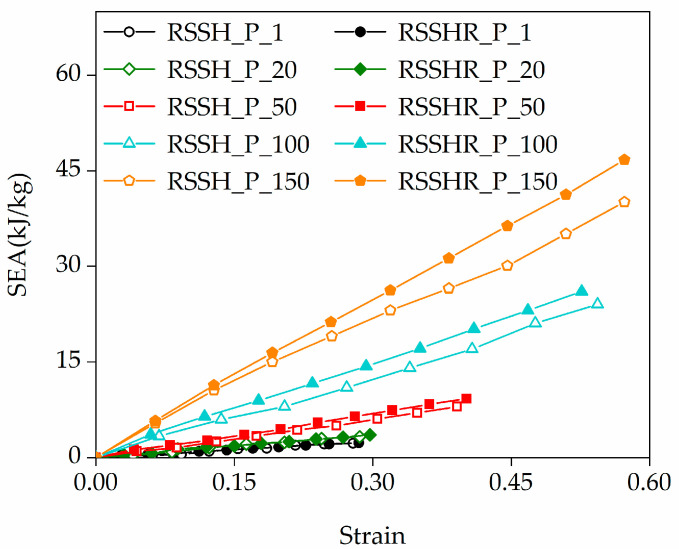
The SEA–strain curves of the RSSH_P and RSSHR_P structures under different crushing velocities.

According to Equation (4), the SEA of the RSSH_P and RSSHR_P structures is calculated. Figure 11 shows the SEA–strain curves of the RSSH_P and RSSHR_P structures when the crushing velocities are different. The solid symbols and hollow symbols represent the RSSHR_P structure and RSSH_P structure, respectively. Symbols of the same shape represent the same crushing velocity. The numbers in the legend are the crushing velocities. It can be seen from Figure 11 that the SEA of the two types of structures increases gradually with the compression strain. When the crushing velocity is a constant value, the RSSHR_P structure exhibits a higher SEA than the RSSH_P structure. Moreover, this advantage becomes more pronounced at higher crushing velocities. For example, the SEA of the RSSHR_P structure is 18.6% larger than that of the RSSH_P structure when the crushing velocity is 100 m/s and the compression strain is 0.4. This indicates that introducing an arc-shaped structure into the RSSH primitive unit cell can enhance the energy absorption capacity of the structure. Therefore, the RSSHR_P structure can absorb more energy. Furthermore, we also find that for the same type of honeycomb structure, SEA increases relatively slowly with the crushing velocity in the lower range (the black, green and red lines shown in the figure), resulting in insignificant differences. In contrast, within the higher velocity range (the red, cyan and yellow lines shown in the figure), SEA increases significantly with the crushing velocity at the same compression strain.

In addition to the above studies, the influence of the radius of the arc-shaped structure on the SEA of the RSSHR_P structure is also explored when the crushing velocity is 10 m/s. The results indicate that the SEA is largely insensitive to variations in the radius of the arc-shaped structure, as shown in Supplementary Figure S4.

## 7. Conclusions

A novel RSSHR primitive unit cell is developed by introducing an arc-shaped structure into the RSSH primitive unit cell. Based on theoretical models and finite element methods, the dynamic mechanical responses and energy absorption capacities of RSSH_P and RSSHR_P structures are investigated. The relationships among NPR, specific energy absorption (SEA), deformed shapes and crushing velocities are elucidated.

The results show that as the crushing velocity increases, the plateau stress and SEA of the RSSH_P and RSSHR_P structures increase. The absolute value of the NPR decreases. During deformation, the gradual engagement and frictional interaction between the re-entrant region in the RSSHR primitive unit cell and the arc structure provide an alternative load path. This mechanism effectively alleviates stress concentration, stabilizes load transfer and promotes more uniform and full deformation of the RSSHR_P structure. The RSSHR_P structure demonstrates superior and stable mechanical performance, particularly under high-velocity crushing. For example, the SEA of the RSSHR_P structure is 18.6% larger than that of the RSSH_P structure when the crushing velocity is 100 m/s and the compression strain is 0.4. In addition, it is also found that at low crushing velocity, the stress–strain curves of the two structures exhibit three distinct stages: the elastic stage (I), the stress plateau stage (II) and the densification stage (III). During the crushing process, there are three deformed shapes. They are the global deformed shape, the local deformed shape and the layer-by-layer deformed shape, respectively.

The primary objective of this study is to evaluate the stability and energy absorption of the RSSHR_P structure under high-velocity crushing. To maintain focus and simplify the analysis, strain rate effects of material and structural parameter optimization are not considered in the simulations. Therefore, future work will not only incorporate strain rate effects and conduct parametric optimization but also include experimental validation of the present simulation results. In addition, the stress-mitigating mechanism of the arc-shaped structure in the RSSHR_P primitive unit cell will be applied to design functionally graded honeycombs which show great promise for applications in aerospace packaging and personal protective equipment.

## Data Availability

The original contributions presented in this study are included in the article/Supplementary Materials. Further inquiries can be directed to the corresponding author.

## Supplementary Materials

The following supporting information can be downloaded at: https://www.mdpi.com/article/10.3390/ma19040716/s1. Figure S1. The influence of the radius of arc-shaped structure on the Poisson’s ratio of RSSHR_P structure. Figure S2. The stress-strain curves of RSSH_P structure when the crushing velocities *v* are (a) 1 m/s, (b) 20 m/s, (c) 50 m/s, (d) 100 m/s, (e) 150 m/s. Figure S3. The stress-strain curves of RSSHR_P structure when the crushing velocities *v* are (a) 1 m/s, (b) 20 m/s, (c) 50 m/s, (d) 100 m/s, (e) 150 m/s. Figure S4. The influence of the radius of arc-shaped structure on the SEA of RSSHR_P structure when the crushing velocity is 10 m/s.
