# Supplementary material for: In-Plane Dynamic Crushing Response and Energy Absorption of a Novel Auxetic Honeycomb
Source: Materials (Basel). 2026 Feb 13;19(4):716. doi: 10.3390/ma19040716 (PMC12942234; doi:10.3390/ma19040716)
Supplement: Supplementary file 1 [file materials-19-00716-s001.zip › materials-4123514-supplementary.pdf]

materials

Article

In-plane dynamic crushing response and energy absorption of a novel auxetic honeycomb

Xin-Liang Li [a,†,*], Bai-Xuan Song [b,†] and Peng Jia [c]

[a]SINOPEC Research Institute of Petroleum Engineering Co., Ltd., Beijing 102206, China
[b]Tianjin Key Laboratory of Modern Engineering Mechanics, School of Mechanical Engineering, Tianjin University, Tianjin, 300072, China
[c]College of Pipeline and Civil Engineering, China University of Petroleum, Qingdao, 266580, China
* Correspondence: author: X.-L. Li; E-mail: lixl@tju.edu.cn
[†]X.-L. Li and B.-X. Song are co-first author as they contributed equally to this work.
B.-X. Song; E-mail: cupid@tju.edu.cn
P. Jia; E-mail: jiapeng2016@upc.edu.cn

1. The Poisson's ratio of RSSHR_P structure

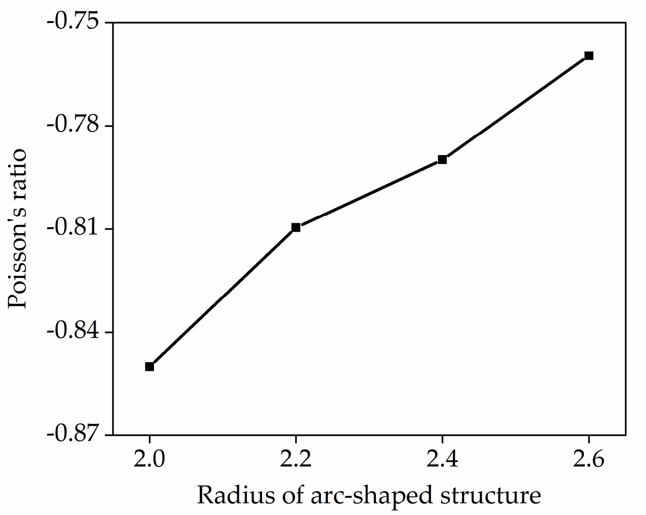

Figure S1. The influence of the radius of arc-shaped structure on the Poisson's ratio of RSSHR_P structure.

Fig. S1 shows the influence of the radius of arc-shaped structure on the Poisson's ratio of RSSHR_P structure when the crushing velocity is 10 m/s. It can be seen that as the radius increases, the absolute value of Poisson's ratio of RSSHR_P structure decreases.

2. Stress-strain curves of RSSH_P structure

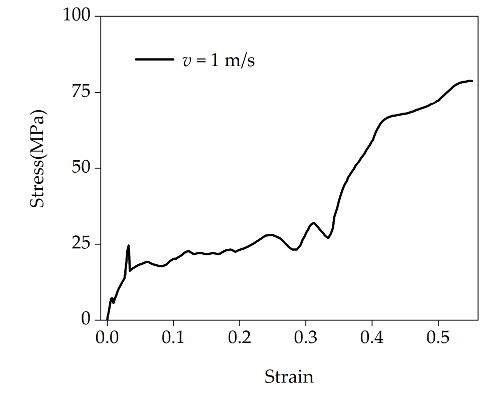

(a) v = 1 m/s

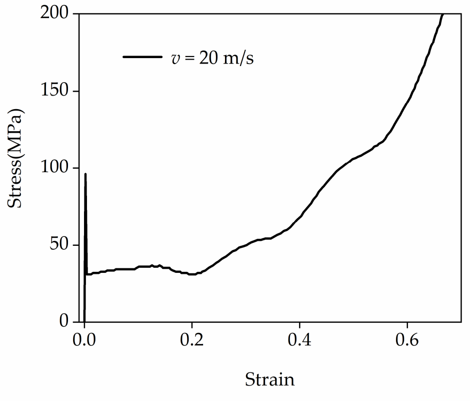

(b) v = 20 m/s

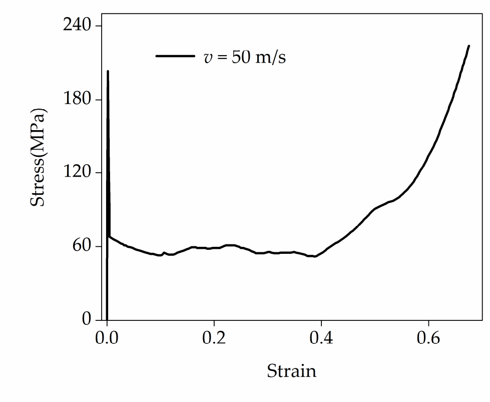

(c) v = 50 m/s

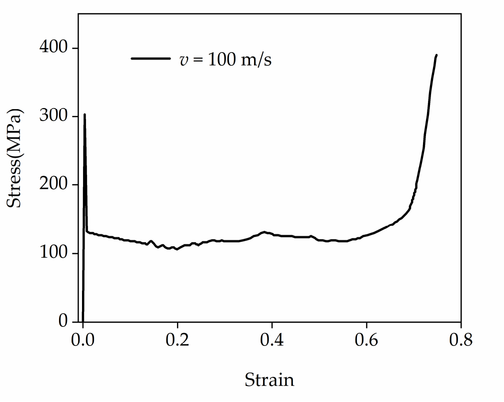

(d) v = 100 m/s

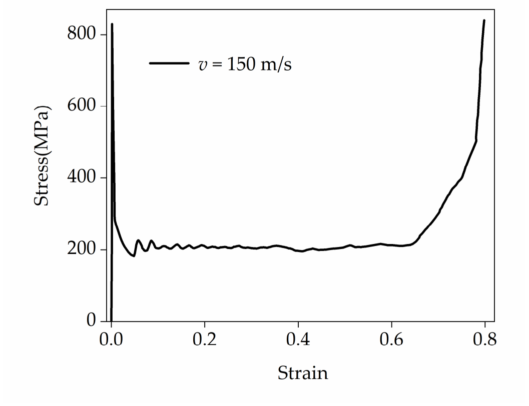

(e) v = 150 m/s

Figure S2. The stress-strain curves of RSSH_P structure when the crushing velocities v are (a) 1 m/s, (b) 20 m/s, (c) 50 m/s, (d) 100 m/s, (e) 150 m/s.

Figs. S2(a)-S2(e) show the stress-strain curves of RSSH_P structure when the crushing velocities v are 1 m/s, 20 m/s, 50 m/s, 100 m/s and 150 m/s.

3. Stress-strain curves of RSSHR_P structure

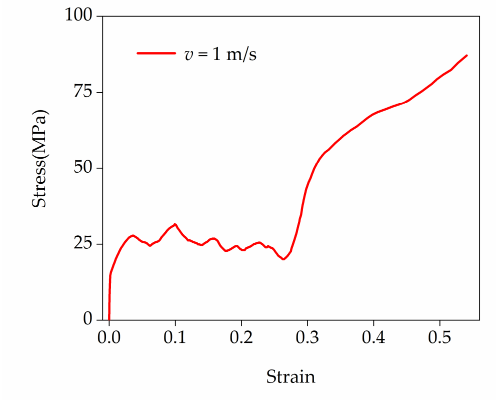

(a) $v = 1$ m/s

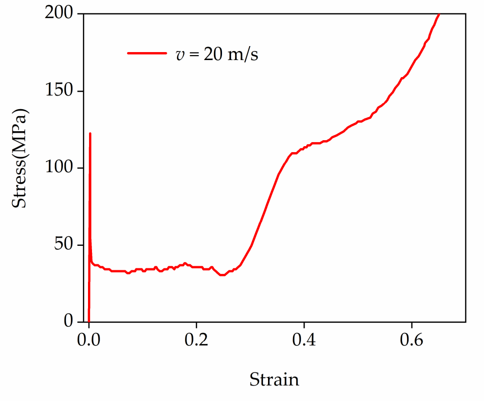

(b) $v = 20$ m/s

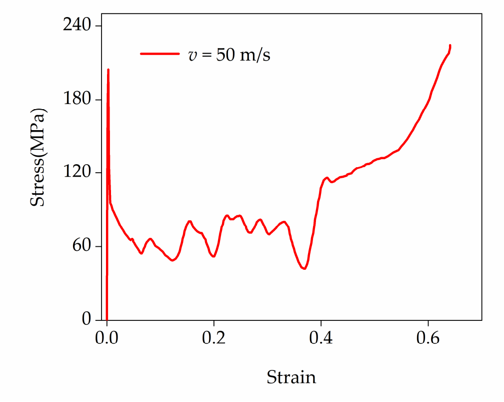

(c) $v = 50$ m/s

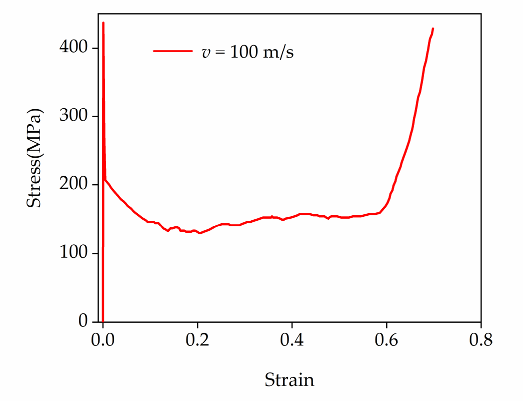

(d) $v = 100$ m/s

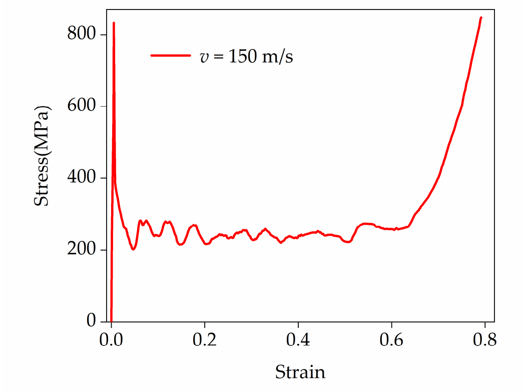

(e) $v = 150$ m/s

Figure S3. The stress-strain curves of RSSHR_P structure when the crushing velocities v are (a) 1 m/s, (b) 20 m/s, (c) 50 m/s, (d) 100 m/s, (e) 150 m/s.

Figs. S3(a)-S3(e) show the stress-strain curves of RSSHR_P structure when the crushing velocities v are 1 m/s, 20 m/s, 50 m/s, 100 m/s and 150 m/s.

https://doi.org/10.3390/ma19040716

4. The specific energy absorption of RSSHR_P structure

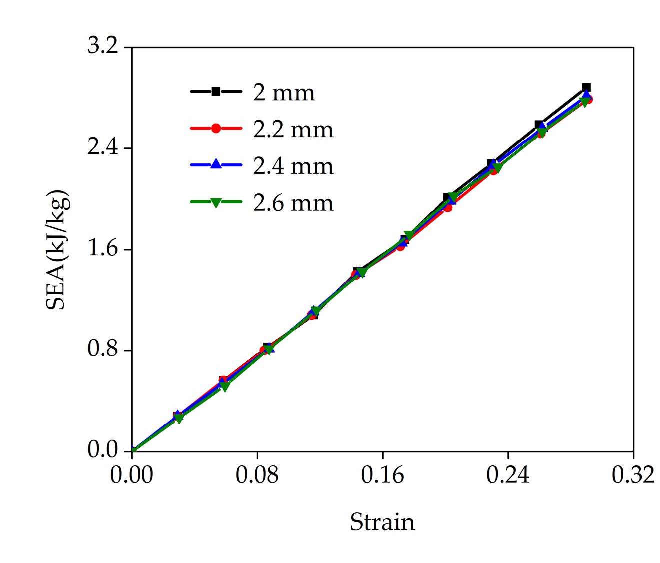

Figure S4. The influence of the radius of arc-shaped structure on the SEA of RSSHR_P structure when the crushing velocity is 10 m/s.

Fig. S4 shows the influence of the radius of arc-shaped structure on the specific energy absorption (SEA) of RSSHR_P structure when the crushing velocity is 10 m/s. It can be seen that the SEA is largely insensitive to variations in the radius of arc-shaped structure.
